# Assessing the integrity of auditory sensory memory processing in CLN*3* disease (Juvenile Neuronal Ceroid Lipofuscinosis (Batten disease)): An auditory evoked potential study of the duration-evoked mismatch negativity (MMN)

**DOI:** 10.21203/rs.3.rs-3203894/v1

**Published:** 2023-08-17

**Authors:** Tufikameni Brima, Edward G. Freedman, Kevin D. Prinsloo, Erika F. Augustine, Heather R. Adams, Kuan Hong Wang, Jonathan W. Mink, Luke H. Shaw, Emma P. Mantel, John J. Foxe

**Affiliations:** 1 The Frederick J. and Marion A. Schindler Cognitive Neurophysiology Laboratory, Department of Neuroscience and The Ernest J. Del Monte Institute for Neuroscience, University of Rochester School of Medicine and Dentistry, Rochester, New York, USA; 2 University of Rochester Batten Center (URBC), Department of Neurology and The Ernest J. Del Monte Institute for Neuroscience, University of Rochester School of Medicine and Dentistry, Rochester, New York, USA; 3 Kennedy Krieger Institute, Baltimore, Maryland, USA

**Keywords:** EEG, Event Related Potential, ERP, Neurodevelopmental Disorder, JNCL, Neurodegenerative disease, Lysosomal Storage Disorder

## Abstract

**Background:**

We interrogated auditory sensory memory capabilities in individuals with CLN3 disease (juvenile neuronal ceroid lipofuscinosis), specifically for the feature of “duration” processing, a critical cue in speech perception. Given decrements in speech and language skills associated with later-stage CLN3 disease, we hypothesized that the duration-evoked mismatch negativity (MMN) of the event related potential (ERP) would be a marker of progressively atypical cortical processing in this population, with potential applicability as a brain-based biomarker in clinical trials.

**Methods:**

We employed three stimulation rates (fast: 450 ms, medium: 900 ms, slow: 1800 ms), allowing for assessment of the sustainability of the auditory sensory memory trace. The robustness of MMN directly relates to the rate at which the regularly occurring stimulus stream is presented. As presentation rate slows, robustness of the sensory memory trace diminishes. By manipulating presentation rate, the strength of the sensory memory trace is parametrically varied, providing greater sensitivity to detect auditory cortical dysfunction. A secondary hypothesis was that duration-evoked MMN abnormalities in CLN3 disease would be more severe at slower presentation rates, resulting from greater demand on the sensory memory system.

**Results:**

Data from individuals with CLN3 disease (N=21; range 6–28 years of age) showed robust MMN responses (i.e., intact auditory sensory memory processes) at the medium stimulation rate. However, at the fastest rate, MMN was significantly reduced, and at the slowest rate, MMN was not detectable in CLN3 disease relative to neurotypical controls (N=41; ages 6–26 years).

**Conclusions:**

Results reveal emerging insufficiencies in this critical auditory perceptual system in individuals with *CLN3* disease.

## INTRODUCTION

CLN3 disease, also known as juvenile neuronal ceroid lipofuscinosis (JNCL – Batten disease), is a childhood-onset neurodegenerative disorder resulting from pathogenic variants in *CLN3* that lead to the pathological accumulation of ceroid lipofuscin in lysosomes of multiple cell types, with neurons displaying particular vulnerability [1] [2]. CLN3 disease is one condition in a genetically heterogeneous class of rare neuronal lysosomal storage disorders, collectively known as neuronal ceroid lipofuscinoses (NCLs). While individually rare, collectively the NCLs constitute the leading known cause of childhood neurodegenerative disorders worldwide [1] [3]. Symptoms typically onset between 4–7 years of age, with progressive neurodegeneration persisting for approximately 20–25 years, leading to premature mortality [4] [5] [6]. The most common initial symptom is a loss of vision that progresses to severe blindness within 2–4 years, which is typically followed by cognitive decline, onset of seizures and Parkinsonism [7] [8] [9] [4]. Throughout adolescence and early adulthood, there is progressive loss of cognitive functioning, speech and motor skills [5] [6] [10]. Because of the combination of progressive vision loss, motor dysfunction, and cognitive decline, it can be challenging to accurately assess the extent of the progressive neurocognitive decline in this population as the disease takes its course, since the administration of conventional cognitive evaluations that require visual presentation of information is not feasible [11]. As such, there is limited knowledge about perceptual and cognitive capabilities across the progressive clinical stages of *CLN3* disease. The consequences of these limitations affect both clinical evaluation and examination of efficacy during clinical trials. There is a pressing need to identify specific quantitative measures of brain function (i.e., neuromarkers or endophenotypes) that could be tracked objectively across the natural course of *CLN3* disease. Such measures would mitigate subjective outcome associated with conventional cognitive evaluation, serve as surrogate biomarkers of disease severity and could provide more precise evidence of treatment effects.

Event-related potential (ERP) recordings are an increasingly appealing option in both human patients and animal models of rare diseases [12] [13] [14] [15] [16] [17] [18]. This easy-to-apply non-invasive technique provides the opportunity to acquire objective quantitative measures of brain activity, including cortical network dynamics, without the need for overt behavioral responses from participants (e.g. [19] [20] [21]), and its exquisite temporal resolution allows for assessment of information flow across the cortical hierarchy, from sensory to perceptual to cognitive stages of processing [22]. Since the peripheral visual system is affected severely and early in *CLN3* disease, primarily due to macular dystrophy [23], here we deployed the ERP technique to measure auditory sensory-perceptual processing as a means to assess the integrity of early cortical processing in *CLN3* disease. This is an important point, since the intention here is to specifically test central cortical processing. As such, the presence of variable peripheral deficits means that visual stimulation cannot be reasonably used to assay the integrity of cortical processing. In contrast, the peripheral auditory system appears to be intact in CLN3 disease, and as such, stimulation can be fatefully delivered to assess central processing dynamics.

The integrity of early auditory processing, auditory discrimination, and sensory memory can be studied by recording the well-characterized mismatch negativity (MMN) component of the ERP [24] [25] . MMN is evoked pre-attentively by introducing occasional changes (deviants) in a regularly occurring stream of auditory inputs, typically by manipulating features such as frequency, location, loudness, phonemic boundaries or duration [26] [27] [28]. MMN experimental designs do not require participant engagement or the ability to follow complex tasks, which makes them ideal for assessment of individuals with limited attention or cognitive impairments. The fact that the MMN has been shown to be generated pre-attentively (automatically) is a key factor in its use in clinical conditions where cognition and attentional functioning is compromised, since its generation does not require engagement with the inputs, and individuals can be engaged in other activities (e.g., reading a book, watching a movie, and even in performing a demanding visual task). A substantial literature has shown that MMN generation to simple feature deviants like “duration” are pre-attentively generated and that attention does not detectably modulate the component [29] [30] [31] [32] [33] [34].

Here, we set out to interrogate auditory sensory memory capabilities in individuals with CLN3 disease, specifically for the feature of “duration” processing, an important cue in speech perception and consequently in task performance [35] [36] [37]. Our primary hypothesis was that the duration-evoked MMN would be reduced in amplitude in CLN3 disease. Based on prior work by our research group using this identical paradigm in multiple other rare disease populations (Rett Syndrome [12]; 22Q11 deletion syndrome [38]; and Cystinosis [13] [39]) and in neurotypical controls [40], we had clear precedence to define both the electrodes where the duration MMN is seen to be maximal over frontal scalp (F3, FZ and F4) and the appropriate timeframe within which to make measurements of its maximal amplitude (~200–240 ms).

An additional design feature of our paradigm was the use of three different rates of stimulation (fast: 450 ms, medium: 900 ms, slow: 1800 ms). This manipulation allows for assessment of the sustainability and robustness of the auditory sensory memory trace, as the amplitude of MMN is directly related to the rate at which the regularly occurring stream of stimuli is presented [41] [42]. That is, when stimuli occur at a rapid rate, the occasional deviants are highly detectable and tend to “pop out” from the background sequence, evoking a robust MMN. As the rate of presentation is slowed, however, the robustness of the sensory memory trace is diminished, the deviant stimulus does not pop out in a highly discriminable manner, and the MMN is reduced or even absent. Thus, by manipulating presentation rate, one can parametrically vary the strength of the sensory memory trace, providing a greater degree of sensitivity for detecting potential auditory cortical dysfunction. Therefore, our secondary hypothesis was that compared to TD controls, duration-evoked MMN amplitude reductions in CLN3 disease would be more pronounced at the slower presentation rates, where greater demand was placed on the sensory memory system.

Finally, since recruitment of participants in rare diseases like CLN3 disease necessitates inclusion of individuals across a large age-range in order to ensure an adequately powered study and to study the progressive stages of the disease, age must also be a consideration in subsequent analyses. It is well-known that auditory responses continue to mature with typical development across age [43] [44] [45], we therefore assessed whether the robustness of the MMN would increase with age in these cohorts.

## METHODS

### Participants

Twenty-five participants with CLN3 disease (i.e., genetically confirmed bi-allelic mutations of *CLN3*) and forty-one age-matched neurologically typically developing individuals (TD) were enrolled. Participants with CLN3 disease were recruited through the University of Rochester Batten Center and TDs were recruited from the local community. The CLN3 disease cohort consisted of 12 females and 13 males, while 16 of the 41 TD participants were male. Four participants with CLN3 disease (3 females; 1 male) were excluded due to excessively noisy EEG data, where less than 50 accepted trials per condition were retained after artifact rejection (see details below). In the case of one additional CLN3 disease participant, where fewer than 50 trials were retained after artifact processing, the ERP data were nonetheless retained in the main analyses due to acceptable signal-to-noise properties (i.e., their evoked potentials did not differ significantly (3 SD) from the group averaged mean waveform). The final cohort consisted of 21 individuals with CLN3 disease (mean age: 16.9±5.5 years; range 6–28 years) and 41 TDs (mean age: 13.9±5.2 years; range 6–26 years). There was no difference in age between TD and CLN3 disease groups (t(60) = −1.39, p = 0.17). All participants with CLN3 disease underwent detailed phenotypic assessment, accompanied by detailed medical history questionnaires completed by their caregivers. All had clinically definite CLN3 disease [46] . Symptom severity was measured using a disease-specific instrument, the Unified Batten Disease Rating Scale (UBDRS) [8] [9] and severity stage was assigned using the *CLN3* Staging System (*CLN3*SS) [7]. The UBDRS includes assessments of physical impairment, seizures, mood and behavior, and functional capability. *CLN3*SS categorizes individuals with CLN3 disease into four stages based on the occurrence of core features of vision loss, seizure onset, and loss of independent ambulation. The lower the score of disease stage (stage 0 – 3), the less severe the symptoms (i.e., individuals in stage 1 have a less progressed disease state compared to those in stage 3). Using the *CLN3*SS, 9 individuals with CLN3 disease were classified in stage 1; 10 in stage 2; 6 in stage 3. Clinical demographics, including age, sex, race, ethnicity, disease stage, age at symptom onset, and medications, are listed in the supplementary materials (Supplementary Table 1). The following exclusion criteria were applied to individuals with CLN3 disease: onset of seizures before 4 years of age, developmental concerns not related to CLN3 disease that occurred before the age of 4, and clear outlier status based on preservation of independent function after the age of 30 years [7]. These criteria did not apply to any individual in our cohort. Other exclusion criteria included uncorrected hearing loss or ear infection on the day of EEG acquisition. Neurotypical (TD) participants were excluded if they had a familial history of a neurodevelopmental disorder, or any self-reported or parent-reported neurological or psychiatric disorders.

### Experimental design

We presented an auditory oddball mismatch negativity (MMN) paradigm while recording electroencephalography (EEG). Experimental procedures were similar to those described in our prior work [15]. Tympanometry was used to rule out middle ear conductive hearing loss in all participants on the day of EEG acquisition. Participants sat in a sound-attenuated and electrically shielded booth (Industrial Acoustics Company, Bronx, New York) on a caregiver’s lap or in a chair/wheelchair. They watched a muted movie of their choice on a laptop (Dell Latitude E640) while passively listening to auditory stimuli presented at an intensity of 75 dB SPL using a pair of Etymotic insert earphones (Etymotic Research, Inc., Elk Grove Village, IL, USA). An oddball paradigm was implemented in which regularly occurring standard tones (STD, 85%) were randomly interspersed with deviant tones (DEV, 15%). These tones had a frequency of 1000 Hz with a rise and fall time of 10 ms. Standard tones had a duration of 100 ms while deviant tones were 180 ms long. The tones were presented with stimulus onset asynchronies (SOAs) of either 450, 900, or 1800ms in separate SOA blocks referred to here as conditions. The order of these conditions (450 SOA, 900 SOA, 1800 SOA) was randomized and each SOA condition block consisted of 500, 250, or 125 trials respectively (supplementary Fig 1A). Participants were presented with 14 blocks in total consisting of 2×450 SOA condition, 4×900 SOA condition, and 8×1800 SOA condition within the experimental session, resulting in 1000 trials per condition. The entire task takes one hour to complete without interruptions.

### EEG acquisition

A Biosemi ActiveTwo system (Bio Semi B.V., Amsterdam, Netherlands) with a 32-channel electrode array was used to record continuous EEG signals. Electrodes were positioned according to the BioSemi Equiradial system, with another 2 electrodes located over the left and right mastoids. The set up included an analog-to-digital converter and fiber-optic pass-through to a dedicated acquisition computer (digitized at 512 Hz: DC-to-150 Hz passband). EEG data were referenced online to an active common mode sense (CMS) electrode and a passive driven right leg (DRL) electrode.

### EEG data processing

EEG data were processed and analyzed offline using custom scripts and routines that included functions from the EEGLAB Toolbox [47] and Fieldtrip toolbox [48] for MATLAB 2016.b (the MathWorks, Natick, MA, USA). The EEG data were first resampled to 128 Hz using the decimate function in MATLAB. The decimate function incorporates an 8th order low-pass Chebyshev Type I infinite impulse response (IIR) anti-aliasing filter. EEG data were then bandpass filtered using a Chebyshev Type II filter with a bandpass set at 1–40 Hz. Continuous EEG data were passed through a channel rejection algorithm, which identified bad channels using measures of standard deviation and covariance with neighboring channels (3–7 channels). Rejected channels were then replaced through spherical spline interpolation (EEGLAB). Data were then divided into epochs that started 100ms before the presentation of each tone and extended to 800ms post-stimulus-onset. Bad trials containing severe movement artifacts or particularly noisy events were rejected if voltages exceeding ±150 μV, followed by a threshold set at two standard deviations over the mean of the maximum values for each epoch (the largest absolute value recorded in the first 500 ms of a given epoch, across all channels for each trial in each condition). The number of accepted trials for each condition and group is presented in supplementary Figure 2. All epochs were then baseline corrected to the 100 ms pre-stimulus interval (−100 to 0 ms). Next, the epochs were averaged as a function of stimulus condition to yield the auditory evoked potential to the standard and deviant tones. To maximize the ERP at frontal sites, the data were re-referenced offline to the left inferior temporal scalp-site T7, or T8 (i.e, the equivalent scalp-site over the right inferior temporal region) if T7 was a noisy channel in a given participant. This approach takes advantage of the inversion of the MMN that is seen between fronto-central and inferior temporo-parietal sites [49] [50]. Finally, we applied de-noising using independent component analysis, usually only removing one or two components reflecting eye-movement-related artifacts following definitions provided by Debener and colleagues [51].

The window for measurement of the MMN was based on four previous studies by our research team using this paradigm [12] [13] [52], where the maximal window for measuring MMN amplitude was found to be between 200 and 260 ms, with a peak typically found between 220–230 ms [12]. We confirmed this timing here by subtracting the grand mean ERP to standard tones from the grand mean ERP to deviant tones (i.e., MMN: STD-DEV). In TDs, the resulting distribution of activity showed a maximal difference at approximately 220 ms (Fig. 2 A–C), fully consistent with the timing seen in this prior work [12] [13] [52]. We then defined a time window of 40 ms centered around 220 ms (i.e., 200 ms – 240 ms) to obtain average MMN amplitudes for every individual and across each SOA. Composite averages generated from F3, Fz, and F4 scalp electrodes were used for further statistical analysis. Please note that in prior work, we have used fronto-central scalp sites (FC3, FCZ and FC4) for these measures, but due to the use of a less dense 32-channel electrode cap in the current work, these fronto-central electrode sites were not available for analysis. However, the MMN is also very well-represented by the nearby frontal scalp electrodes (F3, FZ and F4) [53].

### Statistical analyses

The primary analysis employed linear mixed-effects modeling (LME) and was implemented to analyze electrophysiological and clinical staging scores based on *CLN3*SS, using the *fitlme* function in Matlab based on the restricted maximum likelihood (REML) method. Our analyses included both discrete and continuous data across multiple levels. Advantages of this approach over standard analysis of variance (ANOVA) have been detailed previously [54] [55]. Post-hoc analyses were performed using linear hypothesis testing on linear regression model coefficients (*coeftest*). Mixed-effects models account for multiple comparisons and tested the fixed estimates of Condition (DEV vs STD), SOA (450, 900, 1800 ms) and Group (CLN3 disease vs TD), while participants were characterized as random effects. The first analyses explore the effects of SOA and condition on the two participant groups and their interactions, while accounting for the potential influence of age as a random factor. The effect of age is assumed to vary randomly across individuals in the sample and the effects were measured to account for individual differences in the outcome variables. By treating age as a random factor, the LME model allows for individual differences in the outcome variables that are associated with age to be accounted for. This can help to improve the accuracy and precision of the estimates for the fixed effects of interest (i.e., SOA and Condition), as well as the random effects associated with individual participants. Using Wilkinson Notation [56], the following linear-model expression was used $LME=\left(ERP_{amplitude}∼1+SOA_{data}+{Condition}_{trial}+{Group}_{participants}+\right.\;{SOA}_{data}\;*\;{Condition}_{trial}\;*\;{Group}_{participants}+\left(1+{Age\text{\textbackslash{}}Subjects}_{ID}\right),\;method=\text{″}REML\text{″})$. Next, an LME was implemented to explore effects of *CLN3*SS with electrophysiological MMN ERP amplitudes (DEV-STD) within the CLN3 disease group as a function of SOA $(LME=\left(ERP_{amplitude}∼1+SOA_{data}+{\text{Condition}}_{trial}+{Score}_{participants}+{SOA}_{data}\;*\;{Condition}_{trial}\;*\;{Score}_{participants}+\left(1+{Age\text{\textbackslash{}}Subjects}_{ID}\right),\;method=\text{″}REML\text{″})\right.$.

### Estimating Bayes Factor t-test

As well as using frequentist probability-based statistics, we also used the Bayesian analog of a t-test (*bf.ttest*) as a *post-hoc* approach to allow us to explicitly determine the amount of evidence in favor of the null hypothesis ($H_{0}$: no interaction) [57]. We estimated the Bayes factors $\left(BF_{10}\right)$ using Matlab code adapted from RStudio (R-Core-Team, 2016; the function *anovanBF* in the toolbox *Bayes factor* [58]). We adopted the commonly used Jeffrey-Zellner-Siow (JZS) prior with a scaling factor of 0.707 [59] [60]. Monte-Carlo resampling with 10^6^ iterations was used for the $BF_{10}$ estimation. Subjects represented the random factor. Importantly, this estimation allows us to quantify evidence that our experimental factors and interactions explain variance in the data above the random between-subject variations. Standard convention stipulates that any $BF_{10}$ exceeding 3 is evidence in favor of the altsernative hypothesis $\left(H_{1}\right)$, while below 1 is in support of the null hypothesis $\left(H_{0}\right)$, and $BF_{10}$ ranging between 1–3 is taken as weak evidence [61]

Finally, using Spearman correlation analyses with bootstrap resampling [62] it was possible to test the relationship between MMN amplitudes and age for each group. To achieve this, data from each SOA were concatenated together in one analysis allowing for more statistically robust examination of MMN maturation across SOAs [63]. However, each individual linear model fit was overlaid to show relationships for each SOA, while concatenated group data 95% confidence bounds were used. Correlations resulting in significant p-values were quantified using Robust Correlation [62]. This approach stringently checks for false positive correlations using bootstrap resampling, including six additional validation tests. Due to the limited sample size and the unequal age distributions within age categories, the bootstrap method was chosen as the most appropriate test to explore the linear relationship between age and clinical severity, rather than including age in the LME model. The bootstrap method allows for random resampling of the original dataset, which creates multiple simulated datasets with replacement. This resampling method can help to address the issue of unequal age distributions and improve the accuracy of the correlation estimate. Moreover, the robust correlation method is particularly useful in situations where the data may contain outliers or have non-normal distributions, which can potentially bias the correlation estimates. This method uses robust estimation techniques that are less sensitive to extreme values and non-normal distributions, which can help to produce more accurate and reliable results [64] [65].

A secondary exploratory analysis was also planned to more thoroughly explore the rich spatio-temporal dynamics of the entire data matrix. Nonparametric cluster-based permutation (see Figure. 5) was employed [66] [25]. By clustering neighboring channels expressing the same effect, this test controls for issues associated with multiple comparisons while jointly accounting for the dependency of the data. For each SOA condition, a paired samples *t*-test was computed between deviant and standard trials (i.e., MMN: DEV-STD) across each channel-time pair. Significant clusters were defined wherein neighboring spatially connected channels and temporally arranged time-pairs exceeded the statistical threshold of *p* < 0.05 (corrected, *a priori* threshold), and then the sum of the corresponding *t*-values was calculated for each of the resulting clusters (cluster-level statistic, *maxsum*). Next, the critical *p*-value for each cluster was calculated using the Monte Carlo estimate. For each cluster, this involved randomly dividing the data into two subsets and calculating a new summed *t*-value for each iteration. By randomizing the data across the deviant and standard trials (i.e., DEV vs STD) and recalculating the test statistic 2000 times, we obtained a reference distribution of maximum cluster values against which to evaluate the statistics of the actual data. Finally, empirical clusters were considered significant at *p* < 0.05 if their summed *t*-value was smaller than the 2.5^th^ percentile (i.e., less than an alpha-level of 0.05, two-tailed), or higher than the 97.5^th^ percentile of the permutation distribution.

## RESULTS

**Figure 1.**
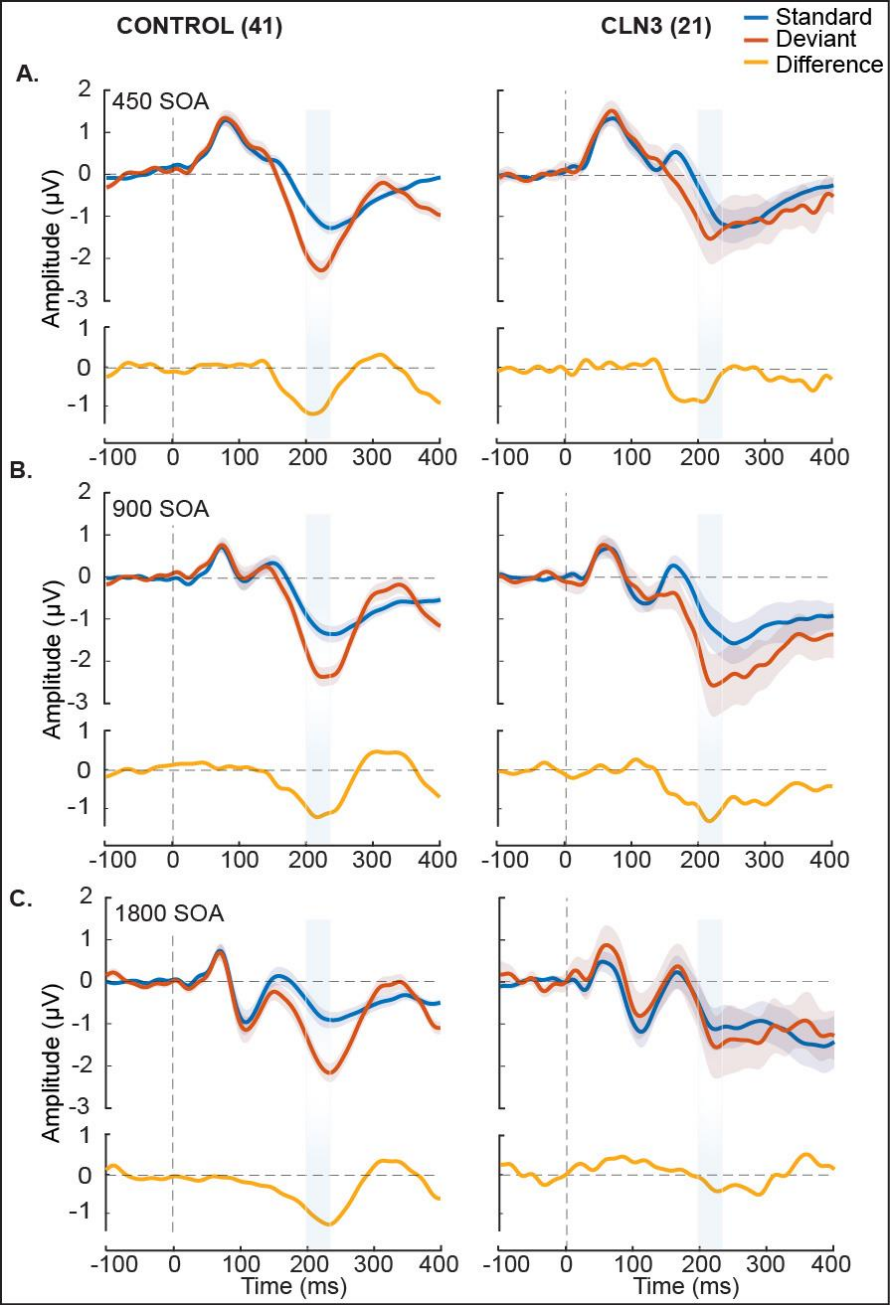
Group-averaged waveforms for typically developing (TD) and CLN3 disease groups over frontal scalp sites (composite average of F3, Fz and F4). Auditory event-related potentials (ERPs) to standard tones (blue trace) and deviant tones (red trace) are presented with standard error of the mean indicated by gray shading. Stimulus onset was at 0ms, indicated by the vertical dotted line. Panel A shows responses for the fastest stimulation rate (450ms stimulus onset asynchrony (SOA)). Plotted in the panel below the ERPs (yellow trace) is the subtraction waveform (deviant minus standard), isolating the MMN-related activity. TD controls are shown to the left of each panel and CLN3 disease individuals to the right. Panel B shows the responses for the medium paced rate (900ms SOA), and panel C shows responses for the slowest rate (1800ms SOA). A clear MMN (difference between standard and deviant traces) was evident at all SOAs for the TD control group. However, a clear MMN was present only for the 450ms and 900ms SOAs in the CLN3 disease group. The time period of interest is depicted by light blue shaded panels representing the defined time where we obtain average MMN amplitudes for every individual and across each SOA.

**Figure 2.**
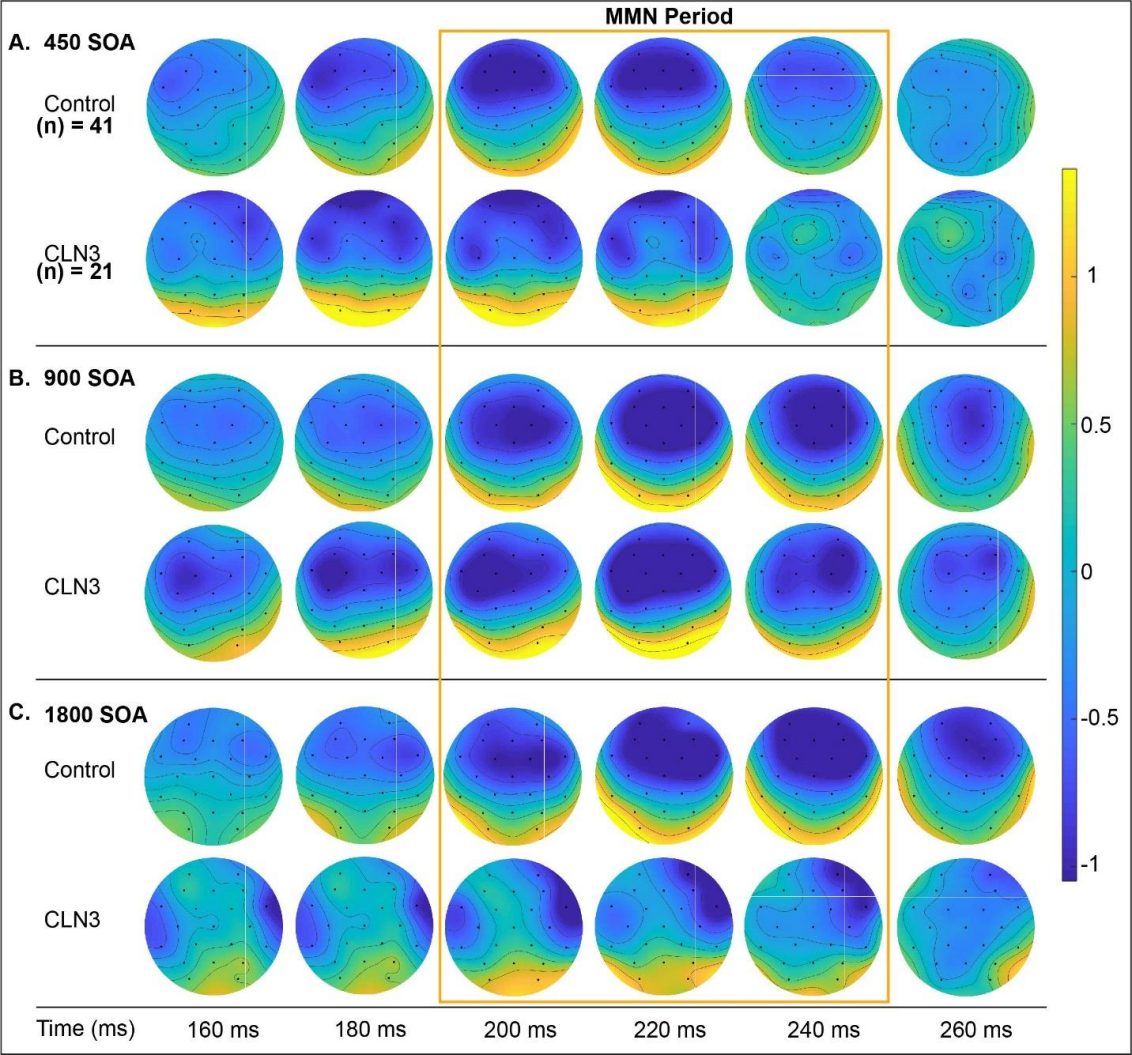
Topographic representation of the differences between deviant and standard tones across SOAs. An MMN with typical spatial distribution with negativity (blue) over fronto-central scalp and positivity (red/yellow) over the mastoids and posterior scalp is clearly seen in all conditions for the TD group. In the CLN3 disease group, the strongest negativity occurs in the 450ms and 900ms SOA conditions, but is substantially reduced with atypical distribution in the 1800ms SOA condition (Panel C)

Figure 1 displays the ERPs elicited by standard and deviant tones for each group as a function of stimulation rate, and the corresponding difference waveforms, over frontal scalp sites (averaged over; F3, Fz, and F4). As expected, TDs show clear canonical MMN responses with a robust negativity in the period from 200 ms - 240 ms post stimulus onset across all SOAs. MMNs to the fast (450 ms) and medium (900 ms) SOA conditions are also evident in the waveform comparisons for the CLN3 disease group, but at the slowest rate (1800 ms), there is no clear MMN in evidence. The MMNs in TDs showed the typical topographic distribution, with a prominent fronto-central negative distribution, accompanied by bilateral positivity over the mastoids (Figure 2), consistent with main generators of the duration MMN in auditory cortices along the supra-temporal plane [49]. Despite weaker magnitudes, individuals with CLN3 disease produced somewhat similar topographical distributions, although at the slowest rate (SOA = 1800 ms), the typical fronto-central distribution was not present (Figure 2, Panel C).

### 1. Modeling effects between groups and stimulus parameters

LME models were implemented to explore ERP amplitudes as the dependent measure, averaged over the time-window of interest (200 ms – 240 ms), and their interrelationships across the two participants groups as a function of SOA and trial type (Condition) as the independent variables, and to test the interactions of these while age and participants were treated as random effects. Outcomes are reported as *beta* coefficients or normalized F1 depending on the model analyses. First, using a multilevel LME comparing all independent variables revealed significant main effects for Group $\left(F_{\left(1,351.69\right)}=6.91.20,\;p=0.008\right)$, SOA $\left(F_{\left(2,326.43\right)}=5.34,\;p=0.005\right)$, Condition $\left(F_{\left(1326.43\right)}=48.71,\;p=1.66\times 10^{-11}\right)$ and Age $\left(F_{\left(1,307.12\right)}=3.51,\;p=0.051\right)$. These results indicate that there was a generalized MMN effect across participant groups. It is noteworthy to mention that conducting a likelihood ratio test that included age as a beta covariate provided a better fit for the data than a model without it $\left(\chi^{2}\left(4\right)=1390.4,\;p=7.36\times 10^{-10}\right)$. To this end, all LME analyses included age as a covariate as it improved the model significantly. Furthermore, there was a significant difference between the DEV vs STD trial conditions across both participant cohorts $\left(\beta =1.01,\;SE=0.14,\;p=1.39\times 10^{-11},\;CI\;[0.72\;1.28]\right)$; indicating a meaningful relationship between participant groups across Conditions as a function of SOA.

In further exploring the relationship of the fixed effects between CLN3 and TD groups, we found a significant positive relationship between Groups (β=0.46,SE=0.17,p=0.008,CI[0.110.81]). This shows that as the MMN effect increases in the TD group the mean MMN effect in the CLN3 group increases demonstrating a positive relationship between the two groups. However, the absolute group means were significantly different from each other, overall, the mean of the TD group is 0.46 higher than the mean of the CLN3 group. Furthermore, there was a significant relationship between participant Group and SOA $(\beta =0.48,\;SE=0.14,\;p=\left.1.71\times 10^{-10}\right)$. Examination of the full model interactions indicated that there was no significant effect between the 450 ms and 900 ms SOAs between Groups. There was, however, a significant effect between 1800 ms and 900 ms SOAs for the CLN3 disease cohort compared to the TD group using age as a covariate $\left(F_{\left(1,155.15\right)}=33.20,\;p=4.34\times 10^{-08}\right)$. Similarly, there was a significant effect between the 450 ms and 1800 ms SOA $\left(F_{\left(1,173,70\right)}=24.01,\;p=\left.2.18\times 10^{-06}\right)\right.$. These results indicate a robust MMN response across groups as a function of Condition, but also point to differential ERPs as a function of presentation rate (SOA).

Next, the analyses focused on the CLN3 group and explored more specifically for MMN effects as a function of SOA within Group. The model revealed that there was an overall significant relationship between the DEV and STD trials (β=1.26,SE=0.41,p=0.02,CI[0.44−2.07]), showing that on average the DEV trial mean amplitudes were 1.26 larger in amplitude than the STD trials. Furthermore, focusing on the association between SOA conditions, there was a significant difference between the 1800 ms SOA and the 900 ms SOA (β=−0.91,SE=0.44,p=0.04,CI[−1.79−0.02]) but no significant difference between 1800 ms SOA and 450 ms SOA (β=−0.22,SE=0.44,p=0.04,CI[−1.11−0.66]) and finally no significant difference between 450 ms SOA and 900 ms SOA (β=0.15,SE=0.22,p=0.51,CI[−0.35−0.66]). Next, planned comparisons comparing DEV vs STD trial conditions (i.e. the MMN effect) revealed there was a significant MMN effect in the 900 ms SOA $\left(t_{\left(1,20\right)}=\right.\left.1.69,\;p=3.21\times 10^{-04},\;CI\;[0.72\;inf]\right)$. However, there was no significant difference in the 450 ms SOA (p=0.053,CI[−0.01inf]) and no difference in the 1800 ms SOA (p=0.23,CI[−051inf]). It was noteworthy though, that in the case of the 450ms SOA, the result was on the bound of conventional statistical significance (*p* = 0.053), whereas this was not the case for the 1800ms SOA (*p* = 0.23). As detailed above, we applied complimentary Bayesian statistics as an alternative method to test for a true MMN effect [57] . The Bayesian analog of the *t*-test allows us to explicitly determine the amount of evidence in favor of the null hypothesis $\left(H_{0}\right)$, for details see Methods section. The results revealed that in the 450 ms SOA condition we had a Bayes factor of $BF_{10}=3.71$ which represents moderate evidence for the alternative hypothesis $\left(H_{1}\right)$ as defined by Lee and Wagenmaker [61] . In other words, this indicates that there is moderate evidence in support of a significant MMN effect in the 450 ms SOA condition. We interpret this finding as evidence for an approach to conventional levels of significance. Furthermore, additional cluster-based permutation statistics on spatio-temporal distribution, reported below (section: *Spatio-temporal Statistical Cluster Analyses*), provide additional evidence in support of a true MMN effect in the 450 ms SOA using Monte Carlo statistics. Next, when further exploring the MMN in the 1800 ms SOA, the results revealed a Bayes factor of $BF_{10}=1.51$, which represents anecdotal evidence for the null hypothesis $\left(H_{0}\right)$. This finding indicates that there may not be a true MMN in the 1800 ms SOA condition as there is only very weak evidence to suggest that there is a significant difference between the DEV and STD trials at this slowest stimulus presentation rate (i.e., anecdotal evidence for alternative hypothesis $\left(H_{0}\right)$). Additional exploratory cluster-based statistics, reported below, provide further evidence to suggest that there is no significant MMN effect across channels and time in the 1800 ms SOA. In sum, these findings suggest that in the CLN3 cohort, the 900 ms SOA evoked a robust MMN effect as compared to the other stimulus presentation rates. In the fastest SOA (450 ms), the MMN approached conventional levels of significance, but there was no statistical support for an MMN at the slowest (1800 ms) SOA.

As a comparison we report the same analyses as above (only the frequentist probability-based statistics) but within the TD group. As done before, the analyses explored more specifically for MMN effects as a function of SOA within the TD population. The model revealed that there was an overall significant relationship between the DEV and STD trials $(\beta =1.09,\;\left.SE=0.11,\;p=4.68\times 10^{-21},\;CI\;[0.89-1.31]\right)$, showing that on average the DEV trial mean amplitudes were 1.09 larger in amplitude than the STD trials. Focusing on the association between SOA conditions, there was no significant difference between the 450 ms SOA and the 1800 ms (β=−0.06,SE=0.17,p=0.71,CI[−0.410.27]) and the 900 ms SOA (β=−0.06,SE=0.17,p=0.72,CI[−0.410.28]). Finally, there was no significant difference between the 1800 ms and 900 ms SOA (β=−0.02,SE=0.11,p=0.85,CI[−0.210.18]). These results show that MMN performance was largely similar across all SOA conditions. Next, to explore MMN effects within each SOA condition, planned comparisons were carried out comparing DEV vs STD trial conditions. This showed a significant MMN effect in the 450 ms $\left.\left(t_{\left(1,40\right)}=-6.47,\;p=\right.\;1.04\times 10^{-07},\;CI\;[-1.38-0.72]\right)$, 900 ms $\left(t_{\left(1,40\right)}=-5.95,\;p=5.52\times 10^{-07},\;CI\;[-1.49-0.73]\right)$, and 1800 ms $\left(t_{\left(1,40\right)}=-6.82,\;p=3.39\times 10^{-08},\;CI\;[-1.45-0.79]\right)$ SOA. As expected, these findings show robust MMN effects across all SOA conditions in TD individuals.

Next, an LME was implemented to explore effects of *CLN3* disease stage (given by *CLN3*SS) on electrophysiological MMN amplitudes (DEV-STD) within the CLN3 disease group as a function of SOA. First, a likelihood ratio test indicated that including age and CLN3 disease stage provided a better fit for the data than the model without them $\left(\chi^{2}\left(9\right)=500.06,\;p<0.05\right)$. Using age as a covariate, LME results showed a significant effect between SOA and CLN3 disease stage 1 and 3 (β=1.36,SE=0.66,p=0.04), while there was no significant effect between stage 1 and 2 nor between stage 2 and 3. These results suggest the larger gap between CLN3 disease stages 1 and 3 is able to distinguish between MMN amplitudes in the current patient cohort. Finally, there was no significant interaction between SOA and CLN3 disease stage.

### 2. Testing the relationship between age and MMN as function of SOA across groups

**Figure 4.**
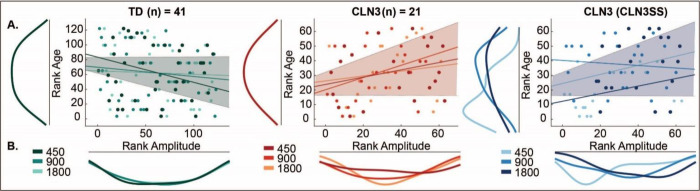
MMN amplitude correlation with Age across SOA conditions. Individual dots represent participants and are color coded according to SOA. Correlations were assessed using robust Spearman’s rank correlation (bootstrap permutation test p < 0.05). This was done for each individual SOA (colored lines), as well as collapsed across all conditions (shaded area representing 95% confidence interval). **A)** Subplots show the distribution of the data in terms of Age (left) and **B)** MMN Amplitude (below). Panel 3 shows ranked *CLN*3SS correlation with MMN Amplitude and Age across SOA conditions.

Robust correlation analysis was used to test the relationship between MMN amplitudes and age for each participant group. The results revealed a significant negative relationship between age and MMN for the TD group $\left(r_{s}=-0.17,\;p<0.05,\;95\%\;CI\;[-0.33-0.01]\right)$, while there was a significant positive relationship between MMN and age within the CLN3 disease group $\left(r_{s}=0.25,\;p<0.05,\;95\%\;CI\;[0.01\;0.49]\right)$. These findings revealed that in TDs, with increasing maturation, the MMN effect becomes stronger. In contrast, individuals with CLN3 disease show that with increasing age, MMN effects become weaker as amplitudes approached baseline (zero). The latter result is not surprising given the age-associated deterioration of both physical and functional capabilities in CLN3 patients that are observed following the initial onset of disease symptoms [8] [7]. Here we repeated the correlation between MMN and age as a function of SOA while data were grouped based on *CLN3*SS (see Figure 4, color coded data). The results revealed a significant positive relationship $\left(r_{s}=0.25,\;p<0.05,\;95\%\;CI\;\left[\begin{array}{ll} 0.01 & 0.4 \end{array}\right]\right)$. These finding corroborate existing literature that demonstrates an age-associated worsening of functional capabilities as well as physical symptom severity in individuals with CLN3 disease [8] [7].

### 3. Exploratory Spatio-temporal Statistical Cluster Analyses

**Fig. 5:**
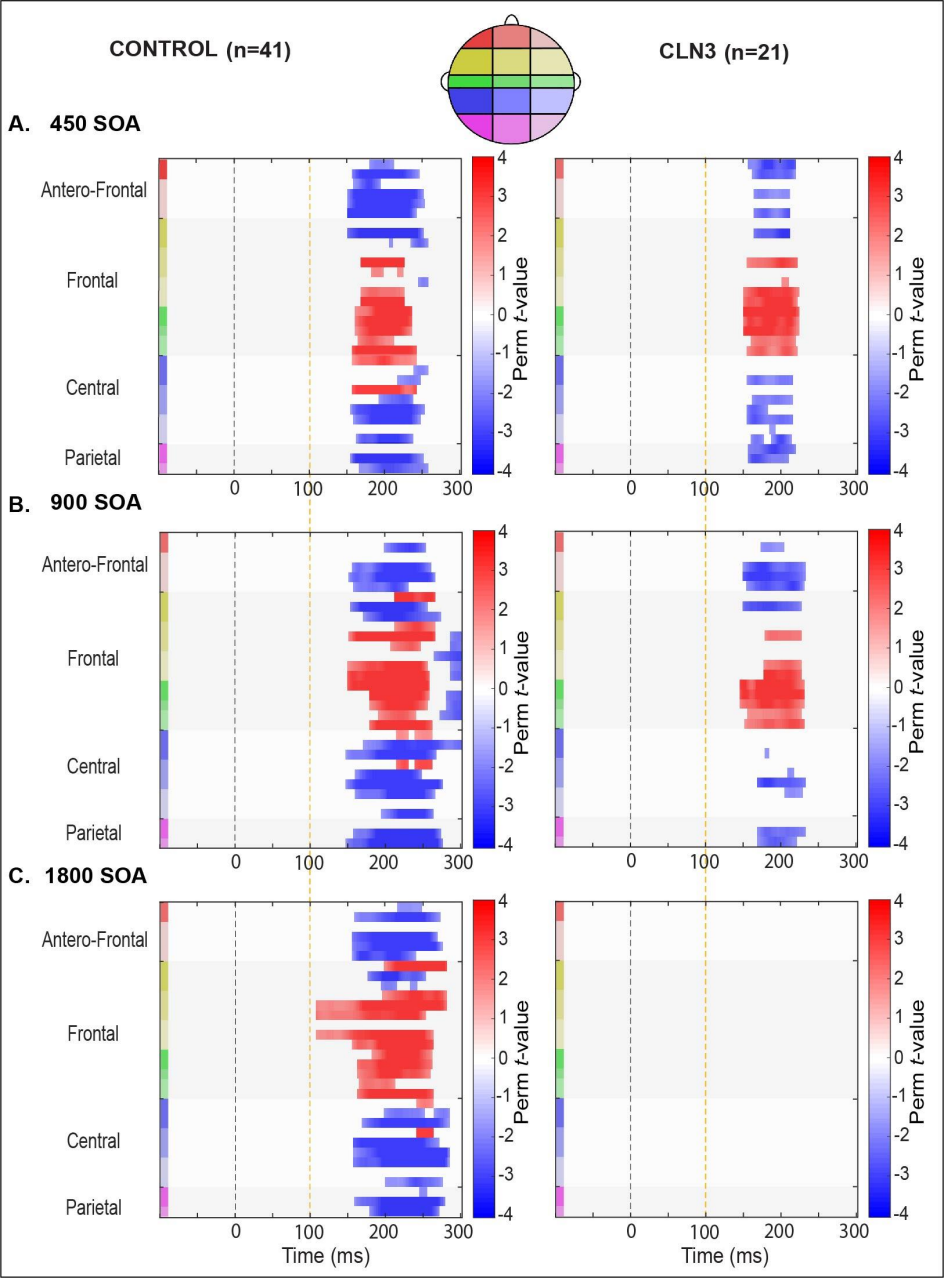
Spatio-temporal cluster-based permutation statistics of MMN across groups and SOA. Nonparametric cluster-based statistics across channels and time, with contrasts between Standard and Deviant tone amplitude differences. Significant clusters are plotted and demarcated by color saturation mask where only significant t-values are shown (p < 0.05, Monte Carlo cluster-corrected, N perm = 2000). The direction of these effects is color-coded, red denotes a positive and blue denotes a negative MMN effect. Time is plotted along the *x*-axis and electrode position is plotted on the *y*-axis. Starting from the top left corner of each graph, electrodes that are located next to each other are clustered into color-coded scalp regions (Antero-Frontal, Frontal, Central, and Parietal). These color-coded regions are displayed on the corresponding head cartoon map (top).

To further explore potentially significant spatio-temporal distributions of task-related activity, an additional exploratory analysis was conducted using cluster-permutation statistics to identify clusters of electrodes and periods of time showing significant differences between standard and deviant tones across SOA conditions. Figure 5 shows the outcomes of this *post-hoc* analysis (*p* < 0.05 corrected, *Nperm* = 2000). For the TDs, there were significant clusters across most channels within the time window of interest 200 ms – 240 ms at all of the SOAs. Contrasts reveal negative magnitudes over fronto-central electrodes. In-line with the ERP waveforms, significant clusters were detected for the 450 ms and 900 ms SOAs at the time window of interest in individuals with CLN3 disease. In contrast, the comparison for the 1800 ms SOA condition showed no clear MMN distribution in the CLN3 disease group at this slowest rate.

## DISCUSSION

The aim of the current study was to utilize the amplitude of the mismatch negativity (MMN) component to assess auditory sensory memory for duration in individuals with CLN3 disease, on the premise that this easy-to-test neurophysiological marker might be sensitive to subtle changes in auditory cortical processing in this progressive neurodevelopmental disorder. Linear mixed effect analysis pointed to an MMN that was intact in the clinical group for medium presentation rates (900 ms SOA), reflecting a generally preserved ability to discriminate auditory duration deviance and to establish auditory sensory memories. However, the MMN was quite clearly compromised at longer (slower) presentation rates (i.e., the 1800 ms SOA) as greater demand was placed on the sensory memory system, in line with our main hypothesis. Results also suggested that at the most rapid stimulation rate (450 ms SOA), the MMN was weaker in the CLN3 cohort, an effect that was not predicted. Finally, we found that age significantly predicted neurophysiological correlates of sensory memory in CLN3 disease – that is, that the MMN showed a progressive reduction in amplitude with increasing age (i.e., disease progression), exactly opposite to what was observed in TD control participants.

In what follows, we describe these results in more detail. First, there was a significant positive relationship between TD and CLN3 participants demonstrating that overall neurophysiological responsivity was relatively comparable across groups as a function of condition, although the absolute group MMN means were significantly different between each cohort. In addition, the *a priori* hypothesis that age would be significantly related to the MMN effect was supported as the overall fit of the model improved when age was added as a covariate. Additionally, there was a main effect for DEV vs STD trials which further demonstrated that there was an overall generalized MMN effect across both participant groups. A key finding was that presentation rate (i.e., the variation in SOA) was a significant predictor of participant group, indicative of an interaction between groups as a function of rate of presentation. When exploring this further using age as a fixed effect, the results showed that there was only a significant difference between the fast and medium SOAs (i.e., 450 ms and 900 ms), but not between the fast and slow SOAs (i.e., 450 ms and 1800 ms) or slow and medium SOAs (i.e., 1800 ms and 900 ms). This suggested that the MMN in CLN3 participants was equally disrupted in the fast and slow presentation rates and the largest gap in auditory sensory memory performance was between the fast and the medium presentation rates.

Next, using a within-subjects model to explicitly test for the MMN effect in the CLN3 group, no significant difference between DEV vs STD trials (i.e., MMN effect) was observed at both the fast and slow presentation rates, whereas there was a robust MMN effect at the medium SOA rate. In partial contrast to the LME results, exploratory *post-hoc* cluster statistics did show a significant MMN effect at the fast presentation rate in CLN3, whereas both statistical approaches showed no evidence of an MMN at the slowest rate. This difference in absence and presence of MMN effect at the fastest presentation rate (SOA = 450 ms) is most likely due to the inherent methodological differences in these statistical approaches [67] . The computation of cluster-based statistics takes into consideration adjacent temporal and spatial information and uses cluster-based correction methods to account for multiple comparisons, while the LME approach relies on specific time-windows of interest at a fixed region of interest (i.e., pre-specified electrodes). Additionally, the LME model used age as a covariate. Lastly, it is worth noting that while the MMN was not strictly significant at the 450 ms SOA in CLN3, it did approach conventional levels of significance in the LME (*p* = 0.053) and Bayesian analysis pointed to moderate evidence for an MMN at this presentation rate. When focusing on the TD cohort, the MMN was clearly present and highly stable across all SOAs, as expected.

Prior work has shown that the strength of the MMN is highly dependent on stimulation rate, with reduced MMN responses observed at slower rates [41] [42] [68]. Current understanding of this phenomenon is that the strength of the auditory sensory memory depends on a temporal integration window, such that establishment of a robust sensory memory depends on the presentation of a number of standards within this window, against which the deviant will ultimately be compared. Perceptually, this is very obvious in a design such as the one used here. At rapid rates of presentation (e.g., (SOA of 450 ms)), the duration deviant pops out strongly from the rapid stream of standards, whereas when the rate of presentation is slowed (SOA 1800 ms here), this pop-out is diminished. In extremis, the reader can readily imagine that if the standard tones were presented once per minute or at even longer lags, it would become very difficult to determine a duration deviant relative to these sporadic standards, and this would certainly not be achieved automatically (pre-attentively). The fact that duration MMN is absent at the slowest and most demanding presentation rate here in CLN3 disease, may point to the early stages of a breakdown in automatic detection and integration of these stimuli in auditory sensory memory. It is also worth pointing out here that the presence or absence of an MMN during passive tasks is known to correspond closely with behavioral performance when individuals are asked to actively discriminate the deviants in follow-up behavioral studies. Only deviants that can be discriminated above chance levels are found to also evoke MMN responses [68] [69].

We did not behaviorally assess auditory discrimination abilities of the participants given the associated loss of vision, speech, and motor decline. Many of the participants with CLN3 disease would not have been able to perform the task. Rather, we employed the passive MMN design to assess the evoked neural activity. It will fall to future work to determine what the perceptual and cognitive implications of this breakdown are [10] [11] [70]. However, prior work has shown that weakened ability to sustain information in sensory memory can reflect cognitive deterioration in various clinical conditions [42] [45] [46]. It will also be of significant interest to further investigate the duration-evoked MMN at even slower presentation rates. This may better reveal the extent of this difference, and it remains to be determined whether this difference is peculiar to the feature of duration or if it will also be evident for other basic auditory features such as pitch, loudness and location. Manipulations of presentation rate are not the only way in which the auditory sensory memory system can be parametrically manipulated. Whereas the presentation rate manipulation used here is presumed to test the temporal integration window of the MMN system, the sensitivity of the system can also be assessed by manipulating the extent to which the deviant stimulus differs from the standards. Here, a deviant of 180ms was used against a standard tone of 100ms, which represents a large and highly discriminable duration change known to evoke large amplitude MMN responses in neurotypical controls [50]. By parametrically manipulating the extent of the duration deviance, prior work has shown that the amplitude of the MMN tracks with the size of the difference, such that at small differences (e.g. 130ms versus 100ms), the MMN is highly diminished or even absent in neurotypical controls [50].

There are parallels between the current findings of diminished MMN responses at slower presentation rates and prior work in other rare neurodevelopmental diseases, specifically Rett Syndrome (RTT) and Cystinosis [12] [13]. In Rett participants, for example, the duration-evoked MMN was only detected when stimuli were presented at the most rapid presentation rate of 450 ms SOA, and unlike the CLN3 disease participants reported here, no MMN was evident at the 900 ms SOA, nor at the 1800 ms SOA, suggesting a more severe disease course in this population. Likewise, participants diagnosed with Cystinosis, another of the rare lysosomal storage disorders, produced robust MMNs comparable to those seen in TDs only in response to the fastest presentation rate (i.e., at 450 ms SOA) [13], with clear atypicalities in the MMN at the two slower rates 900 ms SOA and 1800 ms SOA). Taken together, these data suggest that the duration-evoked MMN may be a sensitive measure of disease severity across a number of neurodevelopmental disorders.

An unanticipated finding here was the weakened MMN response in CLN3 disease at the fastest presentation rate (i.e., the 450 ms SOA). This is the rate at which one expects the most robust MMN to be produced, whereas it was at the medium rate (900 ms SOA) that this occurred in CLN3. Since this was not explicitly predicted, the effect warrants replication in an independent cohort before any strong conclusions can be drawn. Nonetheless, these data suggest that there may be an emerging deficit in the ability to generate auditory sensory memories for duration at rapid presentation rates in CLN3 disease.

Clinically, some of the most striking differences observed in individuals with CLN3 disease are in memory, attention and speech functions [5] [6] [10] [11]. This cognitive decline generally begins around the time of onset of vision impairment, but continues to progress over years, even after vision loss is maximal [9] [71]. To date, quantitative characterization of these differences has not been well-defined [11]. As such, the relationships between cognitive impairments and other clinical features of CLN3 disease are not yet well understood. For instance, the onset of visual decline and of cognitive deterioration have been a subject of debate [72]. It is generally accepted that the onset of observable cognitive decline begins within two years of the onset of visual decline [72] [10]. This has been shown in some individuals with CLN3 disease, while in others, this decline seemed to precede visual deterioration or even emerge at a much later stage [73] [74] [75] [76]. These inconsistencies in the manifestation of the onset of cognitive decline were taken to highlight the importance of careful acquisition of patient history in those suspected to have CLN3 disease [72]. Similarly, understanding the extent of cognitive regression in CLN3 disease is an important component in identifying reliable neurophysiological biomarkers of this disease. As far as we know, the use of electrophysiological assays to evaluate cognitive abilities including attention and memory has not yet been leveraged in this population. The current work serves as a good first step in exploring and developing objective neural markers of pathology (biomarkers) that can be easily carried out noninvasively throughout the progressive stages of CLN3 disease.

Genetically manipulated mouse models of disease are remarkably powerful research tools, providing essential insights into the neurobiological substrates of neurodevelopmental disorders like CLN3 disease [77] [78] [79], and yet many of the outcome measures used to quantify or track disease progression in a mouse cannot be meaningfully applied in human. Obviously enough, invasive electrophysiological recordings, ubiquitous in model systems work, are not feasible in humans. Similarly, many of the behavioral assays used to assess disease progression and severity in a mouse are only loosely related to human behaviors [80], and higher-order functions such as cognitive control and language cannot be readily interrogated. Establishing objective neurophysiological markers of disease progression in human patients is a crucial step towards bridging this inter-species translational divide. In humans, measures of brain electrophysiology are almost exclusively made using non-invasive scalp recordings that assay the activity of large distributed neuronal ensembles across the entire brain (i.e., circuit-level analysis). In mouse models, typical assays involve single or multi-unit neuronal recordings in vivo (usually in anesthetized preparations) or in vitro slice preparations where synaptic plasticity can be assessed. Again, while the approaches used in each species are certainly powerful in their own right, the researcher is mostly left to infer or speculate about correspondences across species. However, ERP markers like the MMN can be readily recorded in mice using wholly similar, if not identical experimental procedures [81] [82]. It will be important to determine going forward whether in mouse models of Batten disease, the MMN phenotype seen here can be recapitulated. If so, it will present as an excellent cross-species neuromarker.

### Study limitations

A few limitations of the current study need to be acknowledged. Given that auditory responses continue to mature with typical development [43] [83], the relatively wide participant age-range is a limitation and follow up studies will ideally work within more delimited age-ranges. Of course, given that CLN3 disease is a rare disease, recruitment within restricted age bands is very challenging. In addition, although age was correlated with MMN amplitude in the TDs, it was not associated with manipulations of stimulus rate. This suggests that the differences seen among groups as a function of presentation rate were not affected by age, but rather, represent frank differences in brain function in CLN3 disease. It will be crucial for future work to follow up with parametric studies to assess the limits of the auditory sensory memory system in CLN3 disease for these and other fundamental auditory features (i.e., frequency, duration, location, and loudness) and their implication for higher-order cognitive processing. Future studies should also follow up with the evaluation of the relationship between ERP measures and the four disease stages based on the *CLN3*SS, with more patients representing each disease stage. In this study, exploring the effects of CLN3 disease stage on MMN amplitudes as a function of SOA while controlling for age proved to be the best model for LME analysis. Although including age as a covariate in the LME model improved performance, the outcomes should be interpreted with caution due to the relatively restricted sample size in each of the stages of CLN3 disease. Again, recruitment within restricted disease stages just as with restricted age bands is very challenging given that CLN3 is a rare disease. We did not include biological sex as a variable in our analyses, and this may be of importance in future work given the reported sex differences in symptom severity and progression in CLN3 disease [84] [85] [86] . The current study was not adequately powered to examine this variable (9 females versus 12 males in our CLN3 cohort). It is worth pointing out though that there is no clear evidence for biological sex differences in the generation of the MMN [87]. Lastly, non-invasive recordings such as those conducted here are limited in their ability to shed light on the mechanisms by which CLN3 protein dysfunction leads to auditory cortical processing differences. Work using similar paradigms in murine models of CLN3 disease will be highly instructive in this regard [88] [89] [79].

### Conclusions

This study points to a preserved ability of individuals with CLN3 disease to automatically decode duration deviations in the auditory stream when stimuli are presented at medium presentation rates. Despite this, automatic detection of duration changes was atypical in these individuals when the presentation rate of the stimulus stream was slowed to both the lowest value 1800 ms SOA) and the fastest (450 ms SOA) used in the current study, suggesting that when additional demand is put upon the auditory sensory memory, more subtle atypicalities are revealed. We speculate that this attenuation in the duration of sensory memory might lead to significant implications for different aspects of information processing, task performance and language acquisition. The exact mechanisms underlying this decline, as well as behavioral outcomes, represent important avenues of research to increase knowledge of CLN3 disease and its perceptual and cognitive sequelae. Measures such as these could potentially serve as surrogate biomarkers with the ability to index disease severity and treatment response.

## Supplementary Material

Supplement 1

## Figures and Tables

**Figure 3.**
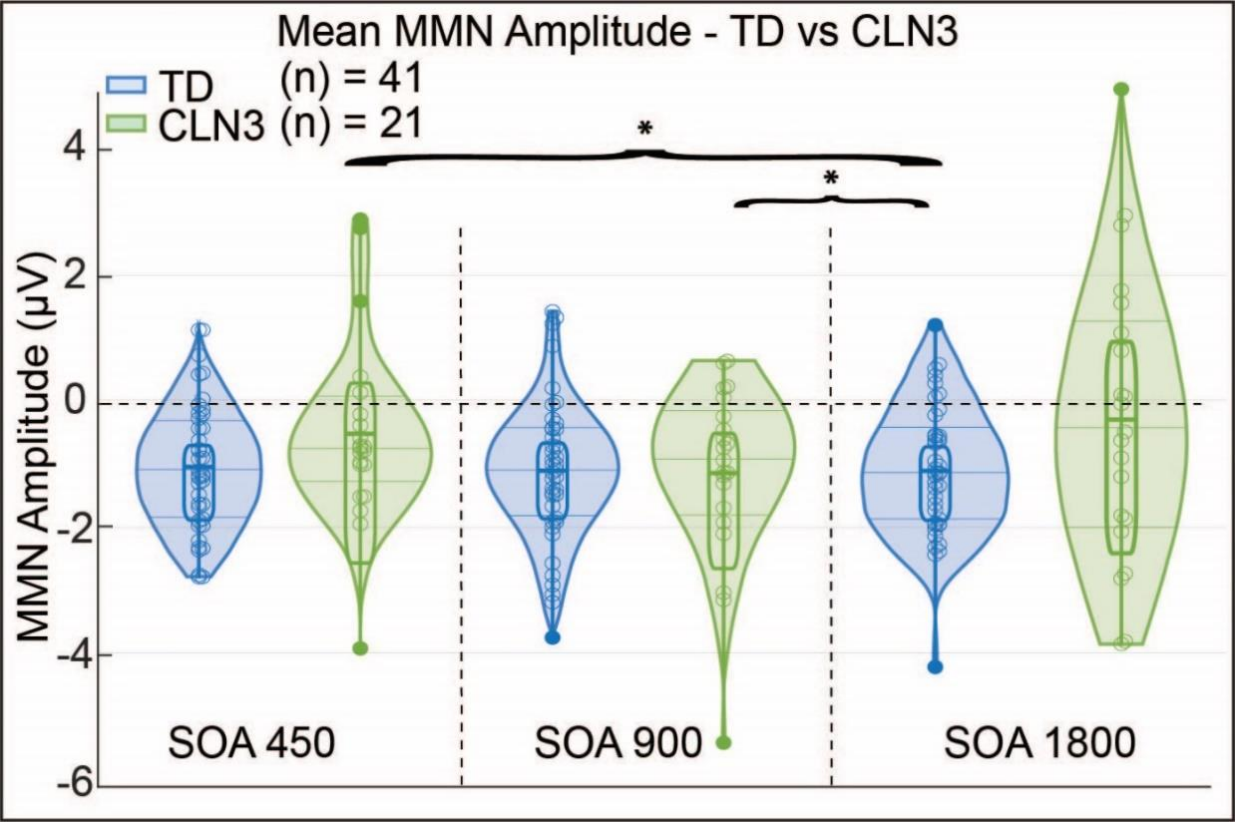
Mean MMN amplitude for each SOA in TD and CLN3 disease groups. Each of the scatter plot, box plot and violin plot columns represent individual participants MMN amplitude values (averaged over; F3, Fz, and F4) as a function of SOA condition. These amplitudes are calculated for the time window between 200 ms – 240 ms. Horizontal lines represent the interquartile range (solid thin lines), median (dashed thick line in box), upper and lower fences that are +/− 1.5 times interquartile range from the median (solid). The blue and green violin plots represent the kernel density estimation for the distributions. Significant effect is between 900 ms and 1800 ms SOA (*p* = 4.34 ×10 ^−08^) and between the 450 ms and 1800 ms SOA (*p* = 2.18 ×10 ^−06^).

## Data Availability

The datasets used and/or analyzed during the current study are available from the corresponding author on reasonable request.

## Abbreviations List:

ERP: Event-Related Potential
MMN: Mismatch Negativity
JNCL: Juvenile Neuronal Ceroid Lipofuscinosis
TD: Typically Developing
EEG: Electroencephalography
SOA: Stimulus Onset Asynchrony
ANOVA: Analysis of Variance
DEV: Deviant
STD: Standard
*CLN3*SS: *CLN3* Staging System
UBDRS: Unified Batten Disease Rating Scale
