# Supplementary material for: Assessing the integrity of auditory sensory memory processing in CLN3 disease (Juvenile Neuronal Ceroid Lipofuscinosis (Batten disease)): An auditory evoked potential study of the duration-evoked mismatch negativity (MMN)
Source: Res Sq. 2023 Aug 17:rs.3.rs-3203894. Preprint. [Version 1] doi: 10.21203/rs.3.rs-3203894/v1 (PMC10462191; doi:10.21203/rs.3.rs-3203894/v1)
Supplement: Supplement 1 [file NIHPPrs3203894v1-supplement-1.pdf]

Supplementary figures and tables:

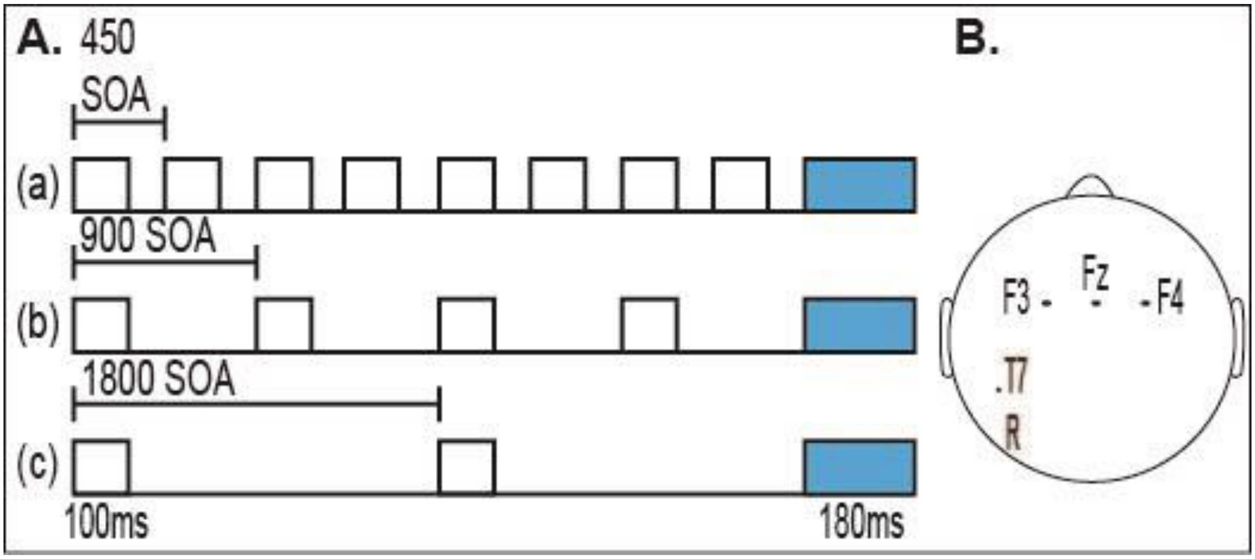

Supplementary Figure 1. Oddball paradigm design. A. Three experimental conditions were implemented, in which auditory stimuli were presented with varied stimulus onset asynchronies (SOAs): (a) 450 ms intervals between stimuli, (b) 900 ms interval between stimuli and (c) 1800 ms intervals between stimuli. B. The electrode sites of interest (F3, Fz, F4) and reference electrode R (T7) are shown on a head map.

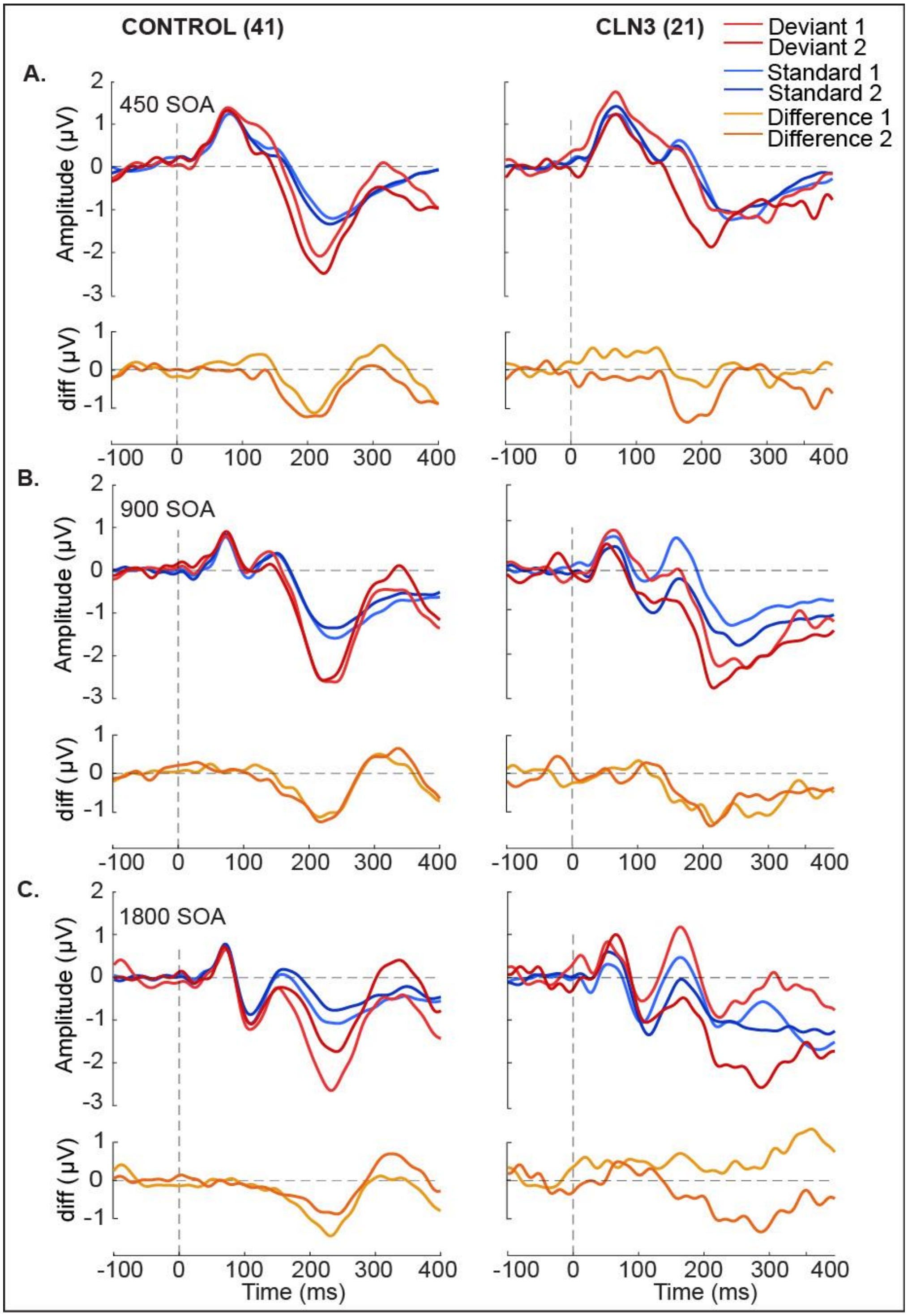

Supplementary Figure 2. Half-split group-averaged waveforms. First half versus second half of the experiment in typically developing (TD) controls and in individuals with CLN3 disease over frontal scalp sites (composite average of F3, Fz and F4). Event-related potentials (ERPs)

to standard tones (light blue trace – Standard 1 represents the first half and the dark blue trace – Standard 2 represents the second half of the experiment) and deviant tones (light red trace – Deviant 1 represents the first half and the dark red trace represents the second half of the experiment) are presented. Stimulus onset was at 0ms, indicated by the vertical dotted line. Panel A shows responses for the fastest stimulation rate (450ms stimulus onset asynchrony (SOA)). Plotted in the panel below the ERPs is the subtraction waveform (deviant minus standard), isolating the MMN-related activity (yellow trace – Difference 1 represents the first half and the orange trace represents the second half of the experiment). Despite the lower number of trials in each condition, the resulting MMN is seen to solidify and improve over time.

Subject	Gender	Age years	UBDRS PS	(*CLN*3SS)	Age of Vision Loss	Age of Seizures OS	Ambulatory	Age of Cognitive R	Medications
1	Male	28	61	3	6 yrs., 2 mon	4 years	No	10 yrs., 0 mon	Trazadone, Depakote
2	Male	17	32	3	5 yrs., 6 mon	6 years	No	8 yrs., 6 mon	Lovastatin, Topomax, Abilify, Lexapro, Depakene, Nexium
3	Male	19	14	2	5 yrs., 6 mon	14 years	Yes	7 yrs., 6 mon	Lovastatin, Ambien, Trazodone
4	Male	20	40	2	5 yrs., 6 mon	10 years	Yes	5 yrs., 9 mon	Copaxone, Topomax, Lovastatin,
5	Female	22	67	3	8 yrs., 0 mon	9 years	No	8 yrs., 3 mon	Trazadone
6	Male	9	3	1	7 yrs., 10 mon	no	Yes	N/A	Lexapro, Lovastatin, Prilosec
7	Female	24	42	2	5 yrs., 9 mon	U	Yes	6 yrs., 9 mon	Lovastatin
8	Male	13	1	1	6 yrs., 5 mon	no	Yes	N/A	Lovastatin
9	Female	10	2	1	9 yrs., 3 mon	no	Yes	11 yrs., 6 mon	none
10	Male	20	N/A	1	7 yrs, 6 mon	no	Yes	7 yrs., 4 mon	Lexapro
11	Female	16	15	1	6 yrs, 0 mon	no	Yes	6 yrs., 10 mon	Lexapro, Copaxone, Trazodone,
12	Female	16	N/A	U	5 yrs., 6 mon	2 years	Yes	11 yrs., 2 mon	Depakene, Lactulose, Lovastatin,
13	Female	17	2	1	5 yrs., 6 mon	N/A	Yes	N/A	Depakene, Lexapro,Lactulose
14	Male	20	N/A	3	8 yrs., 0 mon	N/A	No	8 yrs., 3 mon	none
15	Male	20	58	3	4 yrs., 6 mon	13 years	No	5 yrs., 6 mon	N/A
16	Male	10	8	2	4 yrs., 11 mon	6 years	Yes	5 yrs., 0 mon	Risperdol and Necon
17	Male	6	2	1	5 yrs, 0 mon	N/A	Yes	6 yrs., 0 mon	Depakote, Carintor, Artane, Copaxone
18	Female	16	10	2	8 yrs., 11 mon	10 years	Yes	12 yrs., 6 mon	Depakene
19	Female	14	45	3	6 yrs., 6 mon	10 years	No	6 yrs., 0 mon	none
20	Female	14	1	1	12 yrs., 0 mon	N/A	Yes	N/A	Abilify, Lexapro, Desyrel
21	Female	15	3	2	8 yrs., 0 mon	11 years	Yes	12 yrs., 9 mon	Diastat, Depakene, Carnitor
22	Female	7	2	1	5 yrs., 3 mon	N/A	Yes	1 yrs., 3 mon	Lexapro, Lamictal
23	Male	8	3	2	6 yrs., 6 mon	6 years	Yes	6 yrs., 4 mon	Lexapro, Lamictal
24	Female	15	60	3	6 yrs., 6 mon	6 years	No	8 yrs., 6 mon	Lexapro, Lamictal
25	Female	13	13	2	5 yrs., 0 mon	6 years	Yes	10 yrs., 0 mon	Lexapro, Lamictal

Supplementary Table 1. Clinical demographics of all enrolled participants with CLN3 disease. Data from four participants (Subjects 22-25) in in the shaded cells were excluded from EEG analysis. *CLN3*SS = *CLN3* Staging Score; Seizure OS = Seizure onset; Age of Cognitive R = Age of regression in years (yrs.) and months (mon); N/A = Not Available, U = undefined, UBDRS PS– Unified Batten Disease Rating Scale Physical subscale score.

	TD (n)=41<br>Mean Age 13.9±5.2			CLN3 (n)=21<br>Mean Age 16.9±5.5			TD mean of all accepted	CLN3 mean of all accepted
	**450 SOA**	**900 SOA**	**1800 SOA**	**450 SOA**	**900 SOA**	**1800 SOA**	**Across Conditions**	**Across Conditions**
Avg. Accepted Standard trial ± SD	767<br>±58	742<br>±90	737<br>±115	716<br>±97	722<br>±132	616<br>±192	749<br>±61	684<br>±99
Avg. Accepted Deviant trial ± SD	135<br>±11	132<br>±17	129<br>±20	125<br>±17	128<br>±24	107<br>±33	132<br>±11	120<br>±17

Supplementary Table 2. Numbers of trial included in the analysis across condition in controls and in participants with CLN*3* disease.
